# Characterization of Contusive Spinal Cord Injury by Monitoring Motor-Evoked Potential

**DOI:** 10.3390/biomedicines12112548

**Published:** 2024-11-07

**Authors:** Angelo H. ALL, Ka-Leung Wong, Hasan A. Al-Nashash

**Affiliations:** 1 Department of Chemistry, Hong Kong Baptist University, Hong Kong, China; 2 Department of Applied Biology and Chemical Technology, The Hong Kong Polytechnic University, Hong Kong, China; klgwong@polyu.edu.hk; 3 Department of Electrical Engineering, College of Engineering, American University of Sharjah, Sharjah P.O. Box 26666, United Arab Emirates

**Keywords:** spinal cord injury, contusion, motor-evoked potential, descending motor pathways

## Abstract

This study involves longitudinal neuro-electrophysiological analysis using motor-evoked potentials (MEP) and the Basso, Beattie, and Bresnahan behavioral examinations (BBB) to evaluate moderate mid-thoracic contusive spinal cord injury (SCI) in a rat model. Objectives/Background: The objective of the study is to characterize the onset and progression of contusive SCI over an eight-week period using a clinically applicable tool in an in vivo model. The background highlights the importance of a reliable and reproducible injury model and assessment tools for SCI. Methods: The methods section describes the experimental setup, including randomly assigned rats in three groups: Sham, Control, and Injury (undergoing a moderate contusive SCI using the NYU-Impactor). MEP monitoring and BBB examinations are conducted at baseline and weekly for eight weeks post-injury. Results: The results indicate that the relative MEP power spectral decreased to 11% and 22% in the left and right hindlimbs, respectively, during the first week post-SCI. In the second week, a slight spontaneous recovery was observed, reaching 17% in the left and 31% in the right hindlimbs. Over the following four weeks post-SCI, continuing deterioration of MEP signal power was observed with no detectable recovery. Conclusions: SCI attenuates hindlimb MEP power spectral and reduces locomotion, though the changes in MEP and locomotion exhibit distinct temporal patterns. The MEP monitoring provides valuable insights into the functional integrity of motor pathways following SCI and offer a sensitive and reliable assessment. By implementing MEP monitoring, researchers can track the progression of SCI and evaluate the efficacy of therapeutic interventions quantitatively.

## 1. Introduction

Spinal cord injury (SCI) is a time-sensitive pathology that lacks definitive treatment. The majority of SCIs in humans result from trauma, such as sports injuries, motor vehicle accidents, or falls, mainly occurring in the cervical or thoracic regions.

In 2024, the World Health Organization (WHO) reported that approximately 15 million people globally suffered from SCI [1]. Research by Ding et al. revealed that male individuals and older adults were significantly more affected than females and younger individuals, estimating the incidence of SCI to be 0.9 million, with 20.6 million prevalent cases and nearly 6.2 million total cases living with disabilities [2]. The direct and indirect socio-economic costs of SCI, including acute care, rehabilitation, first year, and yearly costs, were estimated to be substantial [3].

There are five major in vivo models of SCI: contusion (e.g., trauma), compression (e.g., tumor), transection (e.g., penetrating injury), ischemic (e.g., due to major surgical procedure), and focal demyelination (e.g., MS), and the pathophysiology of each model corresponds to a precise pathology in humans. One of the most clinically relevant SCI animal models is the contusive injury, in which a relatively small area of parenchyma quickly absorbs the high energy of impact. Contusive SCI is characterized by disruption and interruption of neuropathways, inflammatory events, apoptosis, and necrosis of neuronal cells, leading to Acute Axonal Degeneration (AAD) and Wallerian Degeneration (WD) [4,5]. One of the hallmarks of contusive SCI is the formation of cavities starting in the Gray matter (the denser part of the spinal cord, where neurons are greatly compacted and, hence, absorb most of the impact energy). The newly formed cavity will gradually and irregularly expand into the surrounding White matter over time [6].

The magnitude of anatomical destruction, and consequently, dysfunction of the spinal cord, depends on the severity of the primary impact, the location of the injury, and the efficacy of the early treatment [7,8,9]. It is evident that SCI results in significant long-term disabilities, including sensory, motor, and autonomic dysfunctions.

SCI is categorized into three phases, though no exact boundaries or time points distinguish one phase from another. The primary injury phase (the first few minutes to a few hours) occurs immediately following the insult to the spinal cord, resulting in vasogenic edema, neural cell death, and disruption of the blood–spinal cord barrier (BSCB). The subsequent secondary injury phase (the first few hours to a few days) involves inflammatory responses, cytokine release, and immune cell infiltration, leading to progressive tissue damage. The tertiary injury phase, extending beyond the first week, involves ischemic injury and further immune cell infiltration, exacerbating tissue degeneration and continuing cell death in the lesion site and surrounding parenchyma [10,11,12,13,14].

The current therapeutic approaches are mostly palliative and include surgical interventions to remove or correct the primary insult and medical treatments that focus on managing symptoms and slowing injury progression [15,16,17]. Emerging pre-clinical treatment modalities and promising clinical studies such as hypothermia [18,19,20,21,22,23,24], stem cell replacement [25,26,27,28,29,30,31,32,33], and functional electrical stimulation (FES) [34,35], in conjugation with long-term physical therapy and rehabilitation [36], are being explored to target specific aspects of SCI and promote recovery at different stages of the injury. Therefore, researchers employ in vivo models to address the unmet treatment needs of SCI.

Somatosensory-evoked potential (SSEP) [37,38,39,40,41,42,43,44,45,46,47,48,49,50,51] and Motor-evoked potentials (MEP) [52,53,54,55] are considered valuable in vivo assessment tools for objectively quantifying the natural history of the injury, evaluating treatment efficacy, and distinguishing between spontaneous and exogenous recovery mechanisms in the spinal cord. While immune-histological examinations and motor behavioral evaluations in rodent models are important assessments, they are considered ex vivo and subjective (prone to human and non-human errors), respectively. This emphasizes the unique value of electrophysiological monitoring of the nervous system. MEP monitors the integrity of the spinal cord motor pathways and motor nerve roots. It measures corticospinal excitability (compound action potential) after axonal stimulation. Characteristically different locomotion among subjects in a group is well-known due to their unique innate motor abilities. Therefore, it is crucial to investigate the neural signature that affects these behavioral differences, which are detectable by reliable measurement of neural activities and a sensitive method to quantify “resting functional connectivity” within the motor neural network.

This article describes a rat model of mid-thoracic contusive injury and neuro-electrophysiological monitoring. The goal is to emphasize the importance of developing precise and reliable tools for tracking and evaluating interventions in SCI research. The article intends to describe the MEP procedure and present a sensitive and specific signal processing method. The objective is to describe how a moderate contusive SCI at the mid-thoracic level precisely affects long-term neural signals and influences functional changes.

## 2. Materials and Methods

### 2.1. Animal Care

All in vivo experiments were conducted in compliance with institutional and governmental regulations pertaining to the ethical and legal considerations governing biomedical research. The surgical procedures were approved by the Johns Hopkins University Institutional Animal Care and Use Committee (IACUC). Data analysis and manuscript preparation were conducted in accordance with the ethical and legal guidelines and regulations of the American University of Sharjah and Hong Kong Baptist University {(23–139) in DH/HT&A/8/2/6 Pt.8}. The experimental procedures adhered to the guidelines set forth by Neuroscience Research and the US-NIH.

### 2.2. Animal Groups

A total of 17 male and female adult (250–300 g) Wistar rats were randomly assigned to the following three groups: (i) Sham group: rats were implanted with the skull screw electrodes but did not undergo laminectomy or contusion (*n* = 5), (ii) Control group: rats were implanted with skull screw electrodes and underwent only laminectomy at T9 (*n* = 5), and (iii) Injury group: rats were implanted with skull screw electrodes, underwent laminectomy at T9, and promptly followed by moderate (12.5 mm) contusion injury (*n* = 7).

### 2.3. Anesthesia

i. It is known that most gas anesthesia drugs, like Isoflurane, induce depression of neuronal activities and decrease the evoked response [56,57,58]. Therefore, we used a mixture of Ketamine (75 mg/kg), Xylazine (10 mg/kg), and Atropine (0.3 mg/kg) during our MEP monitoring. It was established that the intra-peritoneal (i.p.) injection of 0.1 mL of the freshly made cocktail containing Ketamine, Xylazine, and Atropine (7.0–1.0–0.5 mixture) 15 min before recording was the best choice of anesthesia for MEP monitoring. Notably, adding Atropine significantly improved MEP signals because Atropine would substantially reduce the risk of cardiac arrhythmia in anesthetized rats [52,55,59].
ii. We used intraperitoneal injection (i.p.) of 0.2 mL freshly mixed cocktail of ketamine (50 mg/kg), xylazine (5 mg/kg), and acepromazine (1 mg/kg) to induce general anesthesia for the surgical procedures, including skull screw implantation, laminectomy, and contusive SCI [7,8,9].

### 2.4. Addressing Pain

During surgical procedures and MEP recordings, we regularly tested the rats’ responses to noxious stimuli to ensure they did not experience any pain or discomfort.

### 2.5. Skull Implantation of Stimulating Screw Electrode

We have implanted five screw electrodes (E363/20, Plastics One Inc., Roanoke, Virginia, USA—E363/20/2.4/Spc Elec w/screw SS) on the skull (on the corresponding cortices, contralateral to the recording limbs) to stimulate the motor cortex. Two screw electrodes were implanted on the right and left hemispheres 0.2 mm posterior to bregma and 3.8 mm lateral to midline (corresponding to the forelimbs), and the other two on the right and left hemispheres 2.5 mm posterior to bregma and 2.8 mm lateral to midline (corresponding to the hindlimbs). A fifth reference electrode was implanted on the frontal bone. These electrodes made slight contact with the dura mater without compressing or penetrating the brain parenchyma. Electrodes, then, were fixed on the skull permanently with dental cement [7,8,9]. This method of electrode implantation is a critical step for stable long-term longitudinal MEP monitoring, enabling us to stimulate the exact cortical motor pathway coordinates during every monitoring trial.

### 2.6. Laminectomy

Laminectomy is performed to expose an area ~0.5 cm diameter of the dorsal part of the spinal cord. It is done under general anesthesia and is an easy and safe procedure. Rats do not experience any pain or discomfort, nor will they have any form of physical disability because of the laminectomy. A surgical scalpel is used to make a 2–3 cm skin incision on the back. Then, the paravertebral muscles underneath are retracted carefully, T9 is identified, and the lamina (the bony structure on the posterior part of vertebrae) at T9 is removed carefully. The dura mater covering the spinal cord remains intact, which is essential for obtaining reliable MEP signals [52,53].

### 2.7. Spinal Cord Injury

SCI is induced by using NYU-Impactor, which is a well-established method. The impactor consists of a 10 g weight drop rod with an impact surface of 2 mm. When the rod is released from a 12.5 mm height, it induces moderate contusive injury in rats [6,7]. The impactor device is calibrated each time and records the impact trajectory, height, time, and velocity of the impact. This is important to ensure consistency and reproducibility among all rats. The NYU-Impactor was developed by Prof. Wise Young (Rutgers U.). This device is connected to a computer equipped with a specialized program, also designed by Prof. Wise Young, which records various injury parameters such as impact trajectory, height, time, and velocity. After the injury is performed, the paravertebral muscles are sutured in layers, and the skin is closed and disinfected. The surgical site is carefully monitored for bleeding or infection for three days post-SCI.

### 2.8. Post-SCI Care

Rats will continuously be observed for signs of pain and infection. After SCI, rats are kept in a warm place (32–34 °C) for 6–8 h and then moved to their individual cages. Rats will be under observation for any sign of infection (blood in urine or whitish color of urine), bleeding from surgery sites, decline in their physical activity (hiding in a corner of their cage), skin wounds, weight, and fur loss. Rats will have free access to food and water placed on the bottom of the cage. On the day of surgery and the following two days, rats receive two injections of 5 + 5 mL saline per day intradermally on the right and left sides of their back to compensate for dehydration. Rats will be injected with 5 mg/kg intramuscular Gentamicin for five days and 0.3 mg/kg subcutaneous Buprenex (an analgesic well-suited for this type of surgery) for 3 days. Rats’ bladders are expressed manually thrice a day until spontaneous voiding is detected [7,8,9].

### 2.9. Neuroelectrophysiology Monitoring—Motor-Evoked Potentials

MEP is the peripheral nervous system’s response to stimuli induced in the motor cortex or within the motor pathways. Our study records the MEP signals from lower peripheral neuropathways and their corresponding target muscles in response to a well-defined electric stimulation induced within the precise area of the motor cortex after a mid-thoracic moderate contusive SCI. Prior to injury, the MEP signal amplitude varies and can exceed ±4 mV at times. The time latency from stimulus to onset of MEP is approximately 15 ms. After the injury, the amplitude decreases depending on the severity of the injury and can reach down to ±0.4 mV or less with no significant changes to latency. These variations of MEP parameters are proportionally related and correlate with the severity of the injury to the motor pathways. For instance, a mid-thoracic moderate SCI causes disruption and interruption of the spinal neuropathways, resulting in a decrease in the central stimulation of anterior horn cells. Consequently, the sum of stimuli that the targeted muscle (located below the injury site) receives is reduced significantly. Any reduction below the detection sensitivity of such stimuli will cause significant detectable (quantifiable and qualifiable) changes to the MEP waveform.

### 2.10. Stimulation (Via Skull Screw Electrodes)

Bipolar stimuli with a train of pulses (150 pulses = 5 pulses × 30 epochs) at 20 Hz and each pulse duration of 100 μsec were delivered to each one of the four screw electrodes. The simulation intensity was set at a range from 5 mA to 12 mA. This range was determined based on the minimum necessary stimulation (threshold) at which the muscle activity was clearly detectable during the baseline recordings. By definition, the numeric value of the threshold for each recording (in each rat) is determined as the minimum intensity necessary to elicit an observable twitch in the corresponding limb that causes no excessive shaking in the rat’s body, other limbs, and head. The stimulation intensity was established to be 50% above the threshold.

### 2.11. Recording (Via Intra-Muscle Needle Electrodes)

**Figure 1:**
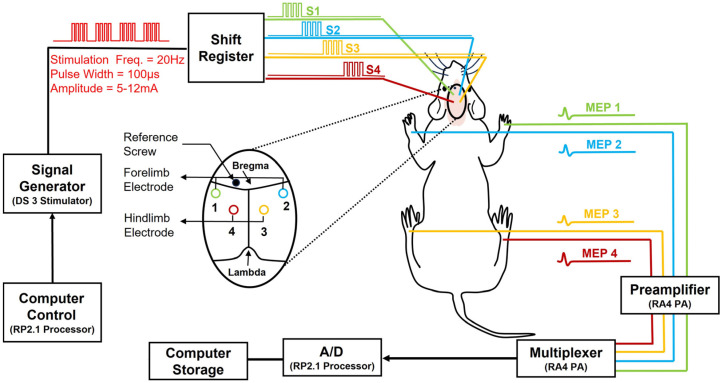
Shows the schematic of the MEP stimulation and the acquisition set-up.

For MEP recording, one needle electrode was inserted into the belly of the tibialis anterior muscle in each hindlimb and one in the extensor digitorum communis muscle in each forelimb. Four other needle electrodes were also inserted into the footpad of each limb as a reference electrode. During each recording session, every 2 s, a pulse train was applied to each skull electrode to obtain a minimum of 30 sweeps from the corresponding muscle. The MEP signals were recorded using an TDT system RA16LI-D low-impedance head stage (Tucker-Davis Technologies System, Alachua, Florida (FL) USA). The MEP signals were sampled at 4882 Hz using an RA16 Medusa Base Station (Tucker-Davis Technologies System, Alachua, Florida (FL) USA) and digitally recorded using OpenEx Recording Software (version 2.32) on a PC. Afterward, MATLAB was used to import the raw MEP signals. The MEP recordings were specified with a bandpass filter with a bandwidth of 10–500 Hz and an analog-to-digital converter. Figure 1 is a schematic of the MEP monitoring set-up system. Four successive pulse trains (S1–S4; Freq. 20 Hz, pulse width 100 μs, amplitude 5–12 mA) were generated for stimulating screw skull electrodes. Four pairs of subdermal needle electrodes were used for the four-channel bipolar MEP recordings. Signals were amplified and digitally sampled (sample Freq.=) at 4882 Hz.

### 2.12. MEP Signal Presentation

The 60 Hz notch filtering has been used to eliminate the powerline interference. This, in turn, improved the signal-to-noise ratio of the MEP signals. In addition, digital lowpass filtering was implemented using a 4th-order filter with a cutoff frequency of 600 Hz. It is noteworthy that since the MEPs are random signals generated by a stochastic process, the direct application of the Fourier transform is not suitable. Hence, by assembling the average power spectrum of the 30 sweeps, the power spectral periodogram was estimated, and the total power under the curve from 5 Hz to 600 Hz was calculated.

### 2.13. Statistical Analysis

The statistical analysis was achieved using MATLAB R2022a. The MEP signals were recorded before the injury (baseline) and once a week for eight weeks post-SCI. The MEP signals were analyzed using repeated measures analysis of variance ANOVA. In our study, the null hypothesis is that the mean power spectrum of MEP signals on days 7, 14, 21, 28, 42, and 56 post-SCI are the same as the baseline (before SCI). Then, from the obtained data, we calculate the *p*-value.

### 2.14. Motor Behavioral Assessment

The Basso, Beattie, and Bresnahan (BBB) motor behavioral examination assesses the hindlimb movements of a rat in a 90 cm diameter open field. The BBB is a well-accepted and widely used standard examination for locomotion in rodents (rats and mice) with SCI [60,61]. In rats, the BBB values are scored from 0 (no limb or join movement) to 21 (healthy subjects with no movement deficits) and are divided into three distinctive recovery phases: early (score 0 to 7), intermediate (score 8 to 14), and late (score 15 to 21). In our study, all rats were examined before and after screw skull implantation, a day before the injury (baseline), on day 4 post-SCI, and once a week for eight weeks following SCI. In order to obtain consistent and reliable scores, healthy rats (before SCI) with any scores other than 21 must be excluded from the study (in our experiments *n* = 0) [62,63].

### 2.15. Histological Examination

Images from histological examinations represent the anatomical structural changes and cavity formation within the spinal cord tissue post-SCI. We used Haematoxylin and Eosin (H&E) staining to produce images of SCI in T6–T12.

## 3. Results

It is important to note that the BBB scoring, analysis, and signal processing were carried out by trained and experienced individuals other than the one experimentalist responsible for performing the laminectomy and SCI in all rats. These individuals were also unaware of the rats’ injury and assigned groups.

We present the MEP data of the two experimental groups: (i) control (rats with implanted skull electrodes and laminectomy) and (ii) contusive injury (rats with implanted skull electrodes, laminectomy, and moderate SCI). Since no meaningful changes have occurred, the MEP data obtained from the sham group (rats with only implanted skull electrodes) are not presented.

**Figure 2:**
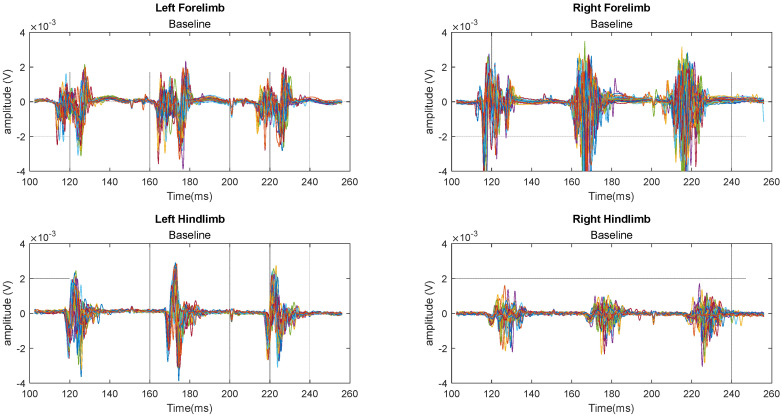
A sample of the baseline MEP time signals showing 30 sweeps obtained from the control group (each color represents one MEP sweep).

Figure 2 illustrates an overlapping 30-sweep time segment of the MEP signals obtained from a representative rat in the control (laminectomy) group during baseline recording. Each of the four signals corresponds to the stimulation of a limb. Notably, each sweep exhibits three MEP activations following cortex stimulations, indicating the rat’s neural response. Following the stimulus onset and the latency period, a prominent downward deflection with a peak exceeding ±4 mV at times is well-detectable. Following this initial peak, additional smaller peaks reflect the complex interactions of stimulated nerve bundles and muscle tissues. Apart from the stimulating smoothed pulse, it is observed that there is no fixed representative MEP wave pattern for all limbs and at different sweeps. The signals are obtained from the left forelimb, left hindlimb, right forelimb, and right hindlimb.

**Figure 3:**
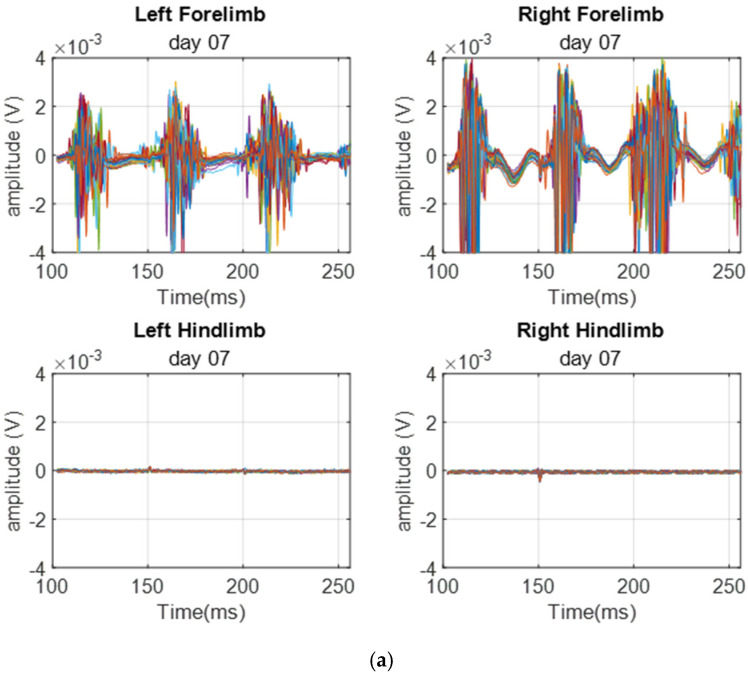

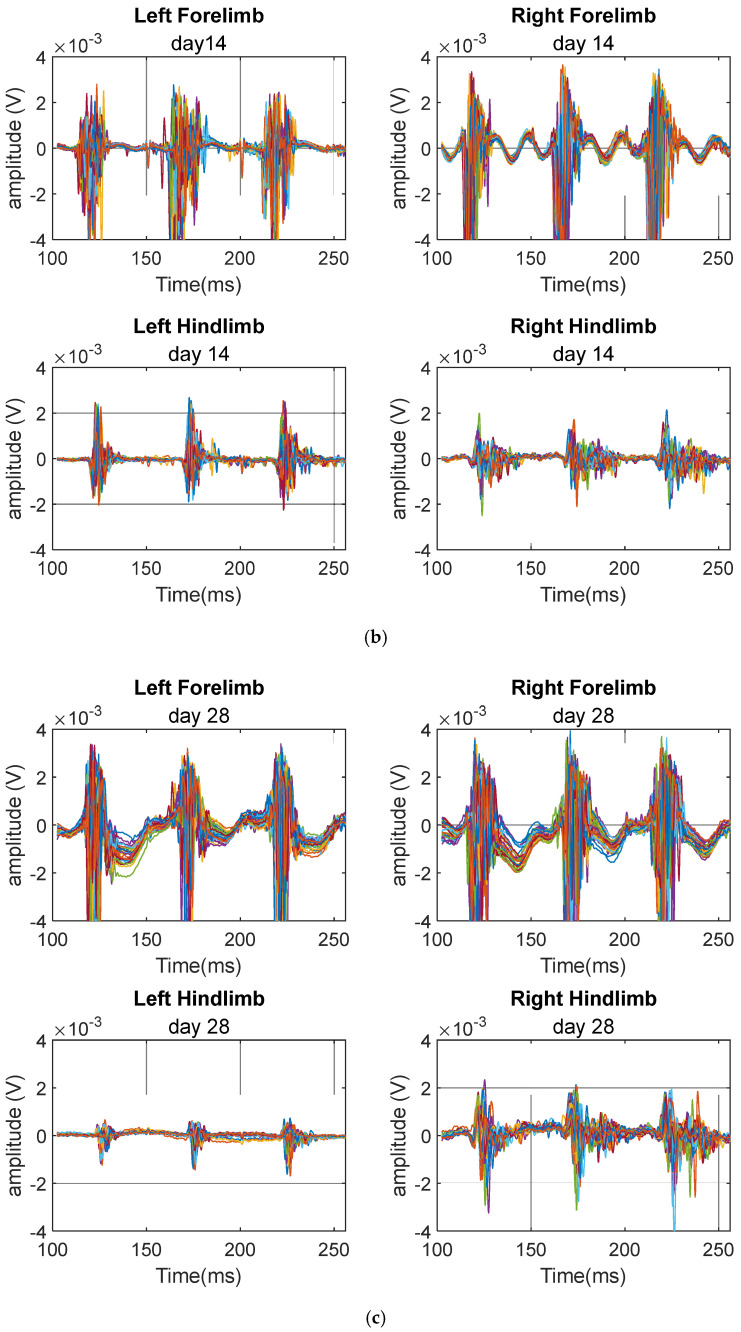
MEP time signals were obtained on (**a**) day 7, (**b**) day 14, and (**c**) day 28 from the same rat in Figure 2.

Figure 3 shows the MEP signals recorded from a representative rat on day 7, day 14, and day 28 post-SCI. The recorded signals show a high degree of visual similarity.

**Figure 4:**
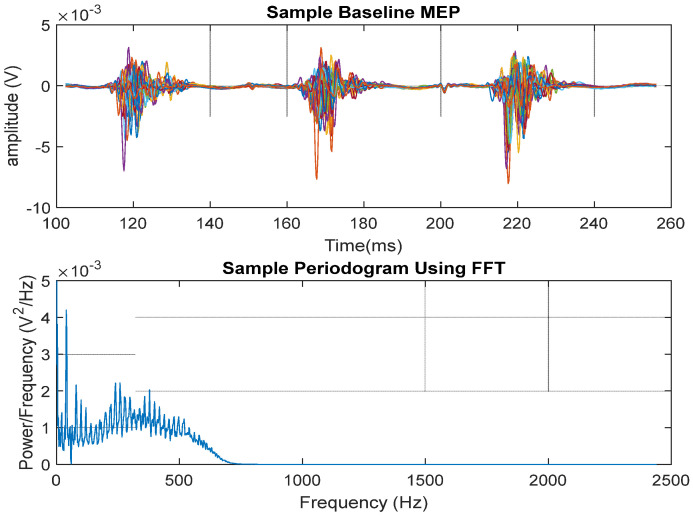
The top shows the MEP time segment obtained during the baseline, and the bottom shows the associated FFT power spectrum density.

Figure 4 shows the MEP time segment (top) obtained during baseline and the associated FFT power spectrum density (bottom). Despite the variations in the power spectrum, a varying power of approximately 1 mV^2^/Hz up to 500 Hz is noticeable.

**Figure 5:**
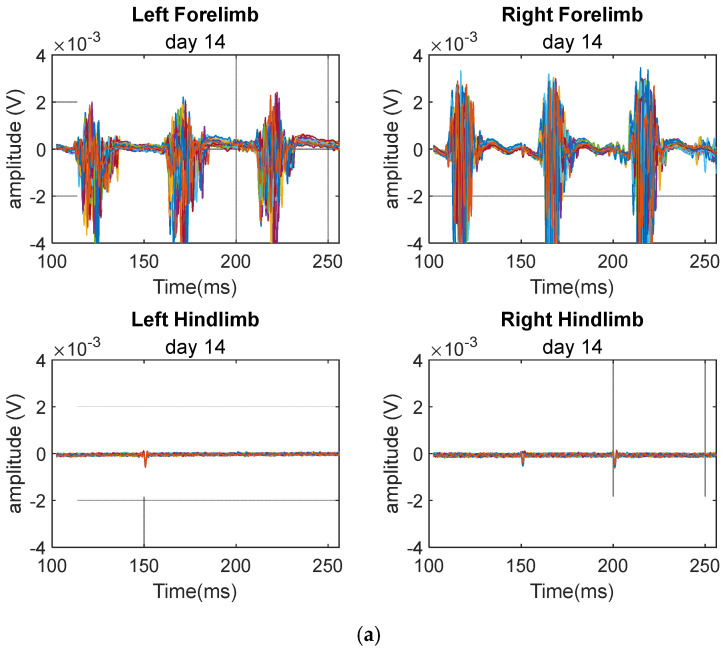

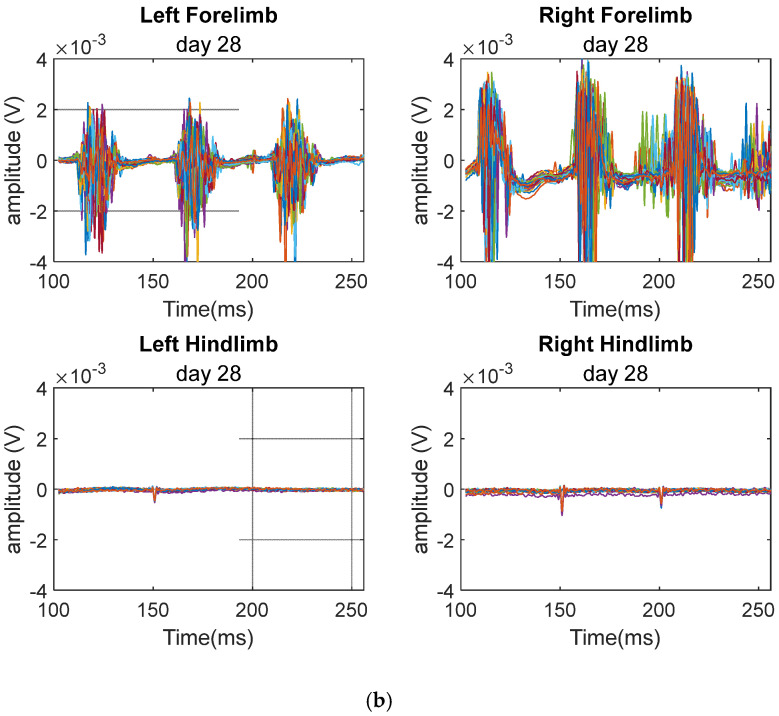
A sample of the baseline MEP time signals showing 30 sweeps obtained from the injury group on (**a**) day 14 and (**b**) day 28. The signals show extreme attenuation to the hindlimb MEP signals.

Figure 5 again shows a time segment of 30 sweeps of the MEP signals obtained from a sample rat from the injury group recorded on days 14 and 28 post-SCI. There are four signals obtained from stimulating the four limbs. As expected, the forelimb signals show patterns similar to those of the control group. However, the hindlimb signals have been severely attenuated.

**Figure 6:**
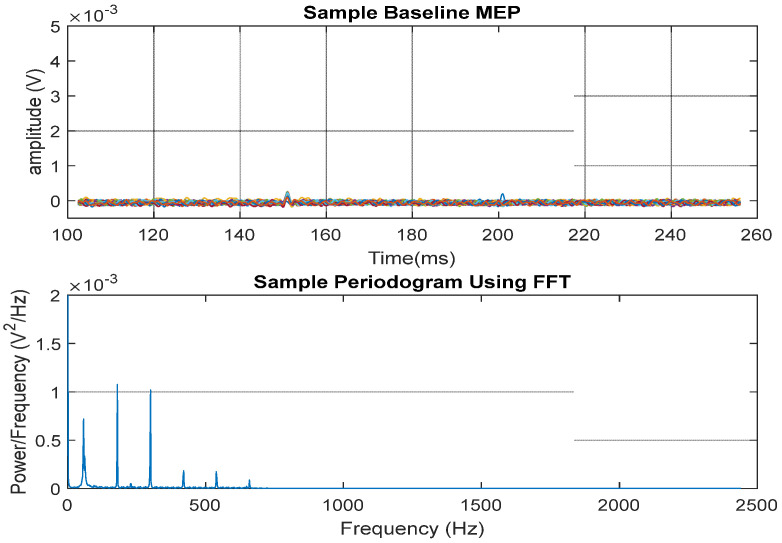
A sample of the sample MEP time signals showing 30 sweeps obtained from the injury group.

Figure 6 shows the MEP time segment (top) obtained post-injury and the associated FFT power spectrum density (bottom). The harmonics shown in the power spectrum are typical for pulsed waveforms (caused by stimulation) with a small duty cycle.

**Figure 7:**
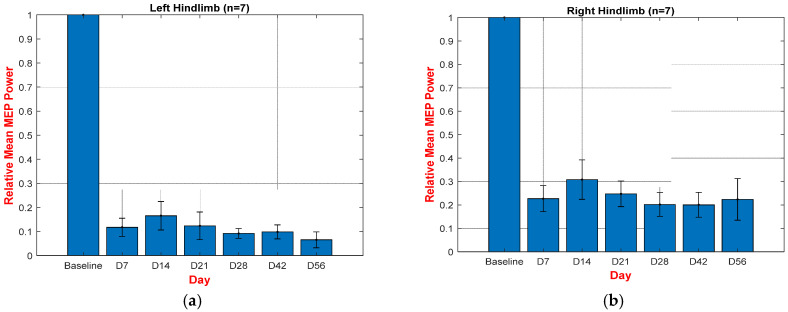
The relative mean spectral power during baseline and four weeks post-SCI in both the left hindlimbs (**a**) and right hindlimbs (**b**).

Figure 7a,b shows the relative mean spectral power during baseline and eight weeks post-SCI in left hindlimbs (a) and right hindlimbs (b). The technical term “relative” here means dividing the mean MEP power spectrum of all seven rats in a certain week post-SCI by the mean obtained during the baseline.

To characterize the degree of functional impairment after SCI, we compared the total spectral power using a repeated measure ANOVA on data obtained from all animals (Table 1). ANOVA calculations allow for collecting data from the same animals on different days, comparing the mean MEP power spectra over time, and assessing their interaction effects. The null hypothesis is that the mean power spectral density of MEP signals on days 7, 14, 21, 28, 42, and 56 are the same as the baseline.

**Figure 8:**
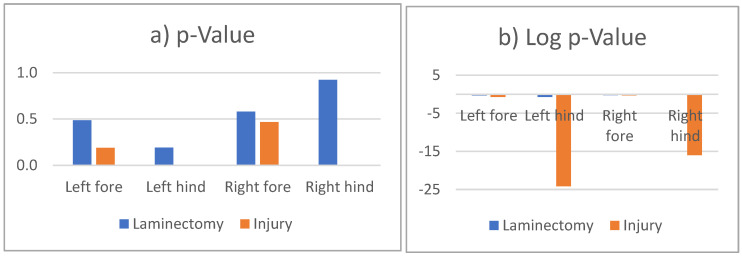
The (**a**) shows the *p*-values and the (**b**) shows the log *p*-Values for the forelimbs and hindlimbs of rats in the control (laminectomy) and injury groups.

From the data, we report the *p*-values shown in Figure 8. Results show that we reject the null hypothesis for the control (only laminectomy) group for both forelimbs and hindlimbs. However, for the injury group, while we reject the null hypothesis for the forelimbs (which is expected), we fail to reject the null hypothesis for the hindlimbs. Table 1 and Figure 8a,b show the *p*-values of the repeated measure ANOVA analysis.

**Figure 9:**
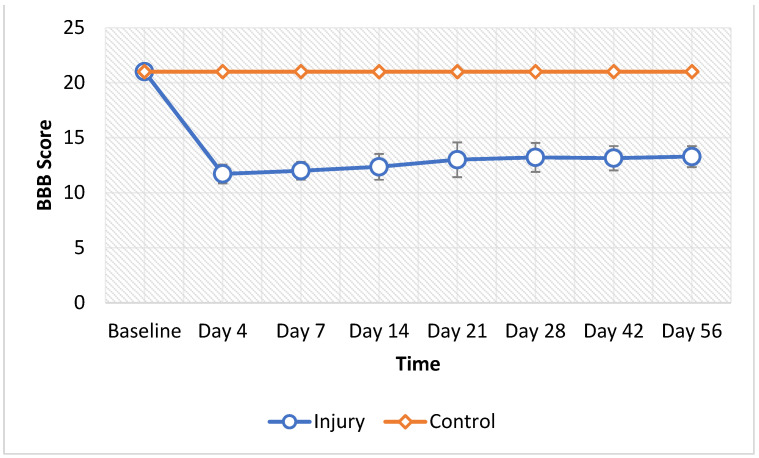
Chronological changes in BBB scores following moderate T9 contusion SCI.

Figure 9 shows the baseline, day 4, and the following eight weeks post-SCI average BBB scores of rats in the control (only laminectomy, no injury) and moderate SCI groups.

**Figure 10:**
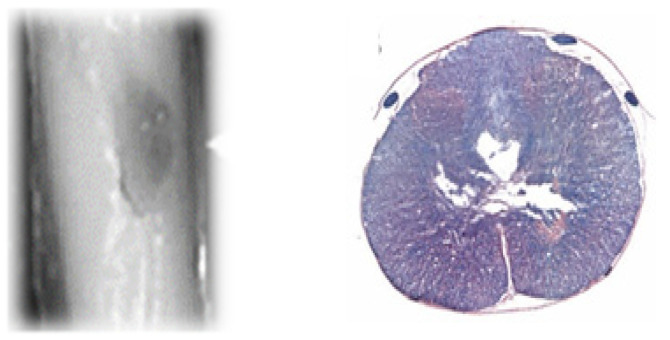
Histological images of the spinal cord post-contusion injury.

Figure 10 shows a hematoma at the site of injury (on the left) and the formation of a cavity within the spinal cord Gray matter post-SCI (H&E on the right).

## 4. Discussion and Conclusions

To evaluate the reproducibility of SCI research models, it is necessary to have reliable, accurate, and cost-effective assessment tools. Neuro-electrophysiological assessments are among objective ‘functional’ assessments of the nervous system, and MEP is an efficient method for assessing SCI. Longitudinal MEP monitoring, at different time points, can accurately detect the onset, characterize the severity, and reveal the progress of injury within descending motor pathways, and can differentiate spontaneous from exogenous improvements post-SCI. Indeed, MEP allows for long-term assessments of neuronal function post-SCI without the need to sacrifice animal subjects.

It is crucial to highlight that BBB scores and MEP signals are not related, and one should not try to find a correlation or connection between them. The early assessment after SCI could explain why there is a difference between BBB scores and MEP values. A contusive SCI almost always causes “spinal shock”, which means that the nerve pathways are not functional during the very early phase of the injury. Moreover, rats do not necessarily need their higher central nervous structures for their limb movements. The spinal cord’s ‘neural circuitry in the ventral horn’, below the injury site, compensates for both voluntary and involuntary movements in rats. Higher BBB scores may also indicate that the spinal cord circuitry is taking over despite the injury to the neuropathways at the lesion site. It is known that rats with as little as 1% preserved and functional neurons can exhibit noticeable lower limb movements. Rats with 10% neuronal functionality show significant motility. In contrast, humans do not show similar compensation, which is attributed to the unique position and function of the corticospinal tract (CST) in rats compared to other mammals. The CST has larger lateral fibers in humans, while there is no dorsal CST in humans compared to rats, who have a large dorsal CST. Both rats and humans have a ventral CST. The CST in rats terminates in different regions of the spinal Gray matter. The CST is the major descending pathway and plays a crucial role in controlling paw movements in rats. Even after SCI at T9 level, the S1 CST response is conserved in rats and its sprouting response indicates an important role in their locomotion [64].

We examined MEP analysis, BBB scores, and histological images of moderate (12.5 mm) thoracic T9 contusive SCI using the NYU-weight drop impactor. The hindlimbs’ MEP signals are attenuated immediately after the onset of injury, down to 11% and 22% of the baseline in the left and right hindlimbs, respectively. Although there was some degree of spontaneous recovery from 11% to 17% and from 22% to 30% in the left and right hindlimbs, due to the nature of moderate SCI (as expected), the MEP signals stabilized mostly after the fourth week post-SCI.

Similarly, the BBB open-field locomotion examinations were performed on day 4 post-SCI and then once a week for eight weeks (and always immediately before the MEP monitoring). Nevertheless, the temporal progression of MEP differed significantly from the BBB score results. This may be attributed to the fact that the muscle responses are not directly correlated to the peripheral nervous system stimulation, which entirely depends on the central (spinal cord) signals. Muscle response often differs from the sum of the maximal electrical input from descending motor neurons. Therefore, the rat’s locomotion BBB scores and the functional assessment of the neuronal pathways must be considered complementary to ensure that changes detectable in the movement ability of the rats have an electrophysiological basis. It is noteworthy that Eidelberg et al. and Loy et al. reported rats’ intact locomotor capabilities even after a complete hemi-transection lesioning the ascending sensory and the pyramidal tracts [65,66]. Some of the divergences between BBB and MEP results could also be due to the re-organization and neuroplasticity of the “spared fibers” within the spinal cord parenchyma in and around the epicenter of the injury [67,68,69,70,71].

In conclusion, our study has provided insights into moderate contusive mid-thoracic SCI induced by NYU-weight drop Impactor using MEP to monitor descending neuropathways and BBB to examine motor behavior over an eight-week observation period. This underscores the significance of incorporating neuro-electrophysiological assessments alongside histological and behavioral examinations in translational SCI research. The distinct temporal patterns observed in MEP and locomotion following moderate SCI emphasize the dynamic nature of SCI and the importance of comprehensive longitudinal monitoring to better detect and identify its progression.

## Figures and Tables

**Table 1:** Shows the *p*-values.

(**a**) Laminectomy						
	**Sum Square**	**DF1**	**DF2**	**Mean Square**	**F**	***p*-Value**
Left forelimb	5.8506	6	24	0.9751	0.9396	0.4854
Left hindlimb	4.2453	6	24	0.7076	1.5972	0.1912
Right forelimb	6.3737	6	24	1.0623	0.7982	0.5807
Right hindlimb	1.3232	6	24	0.2205	0.3172	0.9216
(**b**) Injury						
	**Sum Square**	**DF1**	**DF2**	**Mean Square**	**F**	***p*-Value**
Left forelimb	11.1982	6	36	1.866371	1.558701	0.187609
Left hindlimb	4.7869	6	36	0.797819	169.0465	7.59 × 10^−25^
Right forelimb	6.1898	6	36	1.031638	0.956734	0.467872
Right hindlimb	3.5699	6	36	0.594991	55.50016	1.00 × 10^−16^

## Data Availability

The data reported in this manuscript from experimental and analytical studies are available from the corresponding author upon request.
